# Evaluating the Impact of Chronic Care Model on Smoking Cessation: An Interventional Study

**DOI:** 10.7759/cureus.61512

**Published:** 2024-06-01

**Authors:** Pankaj Chaudhary, Deepika Choudhary, Anshdeep Singh, Salma H Mulla, Prashant GM, Nitin Modi, Priyanka Paul, Kumar Gaurav Chhabra

**Affiliations:** 1 Department of Public Health Dentistry, NIMS Dental College and Hospital, Jaipur, IND; 2 Department of Conservative Dentistry and Endodontics, Seema Dental College and Hospital, Rishikesh, IND; 3 Department of Public Health Dentistry, Al-Ameen Dental College and Hospital, Bijapur, IND; 4 Department of Public Health Dentistry, College of Dental Sciences, Davanagere, IND; 5 Department of Prosthodontics, Vyas Dental College and Hospital, Jodhpur, IND; 6 Department of Public Health Dentistry, Sharad Pawar Dental College and Hospital, Datta Meghe Institute of Higher Education and Research, Wardha, IND

**Keywords:** primary healthcare, socioeconomic factors, urine cotinine level, smoking cessation, chronic care model

## Abstract

Background: This study aims to assess the effectiveness of the chronic care model (CCM) in helping primary healthcare workers quit smoking. The intervention involves implementing the CCM, which includes six key elements: the healthcare system, clinical care planning, clinical management information, self-management guidance, community resources, and decision-making.

Material and methods: The study is based on a population of 60 primary healthcare workers who smoke. The main outcome measure is smoking cessation, determined by cotinine levels in urine at the baseline, and at 6 and 12 months after the intervention. Other potential results include alterations in smoking-related behaviors and attitudes. Data analysis involves using descriptive statistics and inferential tests to determine the intervention's effectiveness in smoking cessation among primary healthcare workers.

Results: The CCM is expected to have contributed to a substantial decrease in the smoking rate among primary healthcare workers. It is also seen that there is a great reduction in urine cotinine levels during the 12-month intervention period. Moreover, a positive shift in the smoking-related behaviors and attitudes of the participants is expected.

Conclusion: This study provides key data about the effectiveness of the CCM in helping primary healthcare workers stop smoking. This statement emphasizes the importance of considering socioeconomic factors in the design and implementation of smoking cessation interventions. This ensures that people of different incomes and social statuses have equal access to quitting smoking and achieve similar results.

## Introduction

Smoking remains a significant public health concern globally, contributing to various chronic diseases and premature mortality. Primary healthcare workers play a crucial role in promoting smoking cessation among patients and the community [1]. The chronic care model (CCM) offers a comprehensive framework for enhancing the management of chronic conditions, including smoking cessation. However, there has been limited research exploring the effectiveness of implementing the CCM specifically for smoking cessation among primary healthcare workers. This study aims to evaluate the impact of CCM implementation on smoking cessation and assess its effectiveness in helping primary health workers quit smoking.

Tobacco use is the primary cause of a significant number of preventable deaths and diseases in many countries. It greatly contributes to various health issues, including but not limited to heart disease, respiratory problems, and cancer [1,2]. Even though many individuals are well aware of the health risks of smoking, quit-smoking campaigns often face significant obstacles when targeting specific population groups, especially primary healthcare workers.

Primary healthcare professionals are actively involved in making the stop-smoking drive successful and are helpful partners for those trying to quit smoking. They have frequent patient contact as frontline healthcare, so they are in the perfect position to develop an evidence-based smoking cessation intervention [2]. Nevertheless, those who work directly with the smoking population may also experience barriers to quitting, such as limited access to cessation resources, inadequate training in cessation counseling techniques, and high levels of stress [3].

The CCM application in primary care seems to be an attractive tool for improving the management of chronic conditions in these settings [4]. Initially, the CCM model aimed to tackle individual and frequently chronic diseases such as diabetes and hypertension. It emphasized patient-centered care, the implementation of evidence-based interventions, and utilizing community resources to support patients in successfully completing their treatment [5]. The literature supports the effectiveness of the model in the treatment of chronic diseases, but smoking cessation interventions are under investigation in this area by specialized healthcare staff.

This intervention aims to address the root causes of smoking behavior and treatment outcomes by taking a comprehensive approach at both personal and systemic levels within the healthcare context. However, the main aim of the study is to evaluate the impact of the Chronic Disease Management Model on cigarette cessation among all healthcare employees. Understanding the effectiveness of the CCM in encouraging smoking cessation among medical staff is crucial to assisting health professionals in designing successful smoking cessation programs. Considering the case of healthcare organizations that can seize the existing resources and infrastructure, there is a strong potential to expand their capacity for quality smoking cessation programs and help improve health outcomes for patients and providers.

## Materials and methods

Study design

Using a longitudinal, interventional study design, we assessed the potential supplements the CCM policy may offer to help primary care providers support their patients' efforts to quit smoking. According to the research, the intervention was applied for one year, beginning with recording baseline data and ending at two predetermined times, 16 and 24 weeks. A volunteer sample of 60 primary healthcare providers who were smokers was included in the study by random sampling. The screening process was conducted based on observing active smoking and job advertisements for primary healthcare roles. Prospective participants were only included in the study group if they had no other medical conditions that would prevent them from taking part in the study; those who stopped participating were also removed from it. The intervention takes the form of "the CCM," a comprehensive treatment framework that includes smoking cessation. The six essential components of the CCM are as follows: the architecture of the healthcare organization and delivery system, decision support, clinical information systems, self-management assistance, and community resources [1,4,5]. Specific intervention components include personalized behavioral counseling determined and directed by the user and establishing follow-up mechanisms to track the participants' pluses and minuses and provide support throughout the trial intervention process [5].

**Figure 1:**
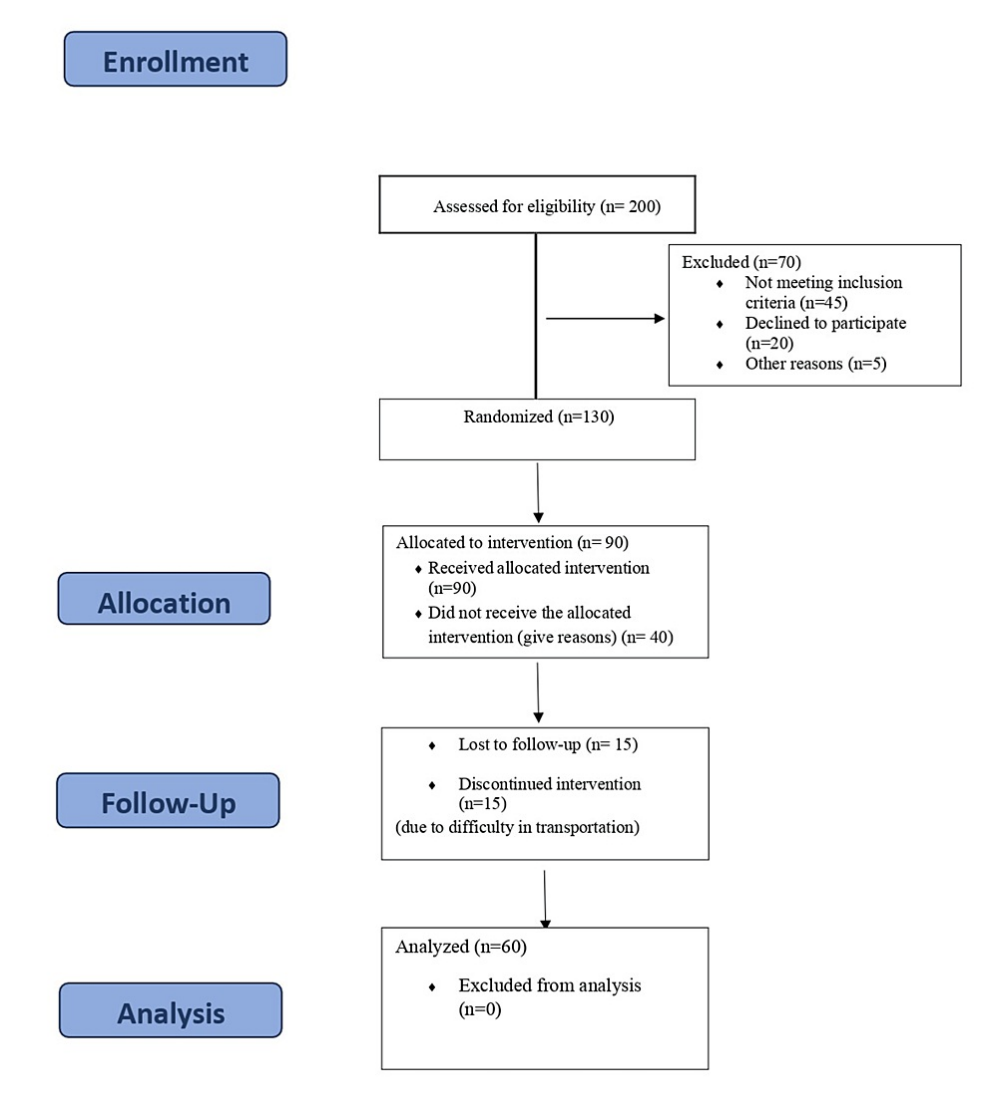
CONSORT flowchart CONSORT: Consolidated Standards of Reporting Trials

Data were collected for the study over the course of three months and one year since the beginning. Demographic information, including age, gender, socioeconomic status, and educational attainment, was collected during the baseline assessment. To confirm the history of smoking, tests for nicotine dependency, including the Fagerström Test, were used. The concentration of cotinine in urine was measured using urine samples as a biomarker. Two investigators were included in the study, and their training for conducting the study was done. Follow-up assessments were conducted at 6 and 12 months, during which the histories and levels of nicotine dependency of the individuals were reviewed. We measured the urine cotinine levels using standard laboratory techniques. Self-reported self-quit outcomes, quit metrics, length of stop, and utilization of cessation services were also collected. The study has received ethical permission from the Institutional Ethical Committee (NIMSUR/IEC/2022/298) of the dental institute, which comprises research experts. All participants' consent was obtained before the study. They understand their involvement in the study. Ensuring participants' complete honesty and confidentiality was a priority during the study period (Figure 1).

Data analysis

This was achieved through descriptive statistics of the means, standard deviations (SDs), frequencies, and percentages in presenting our participants and some of the cigarette-related traits at baseline. A paired t-test was used to evaluate the change in urine cotinine levels over time to monitor the effect of the intervention from the baseline. For example, we also conducted a subgroup analysis to investigate how socioeconomic factors impact the success of quit-approach strategies. In this research, we considered p ≤ 0.05 as the statistical significance. Additionally, we compared the test statistics, such as the t-test or chi-square test, between the groups and presented the results accordingly.

## Results

Participant characteristics

Sixty primary healthcare workers with a habit of smoking had a mean age of 38 ± 5.2. The gender distribution was relatively noted, with 50% male and 50% female participants. The age group had participants with a vice-smoking history of 12 years (12 ± 3.8) and a daily consumption of 15 cigarettes (15 ± 4.5) per day.

Additionally, urine cotinine levels were recorded during the baseline and after six months of the interventions. Furthermore, the mean urine cotinine level was correlated with socioeconomic factors. Each age group exhibited a difference from baseline to six months of age. The mean values were higher in the 46-60-year-old age group, and they were most likely male. Researchers measured urine cotinine levels at the start of the study and again after 12 months of the intervention. The test checked the urine cotinine levels at different time intervals, which have been statistically proven to differ; thus, the likelihood that the difference in the urine cotinine levels between baseline and 6 and 12 months after the intervention was due to the chance of the implementation of the CCM is high. A p value <0.05 is regarded as statistically significant (Table 1).

**Table 1:** Comparison of urine cotinine levels at baseline and 6 and 12 months postintervention, stratified by socioeconomic factors The urine cotinine levels for the study were measured at two time intervals, i.e., 6 and 12 months after the intervention was applied. A statistically significant difference was observed at each time interval.

Demographic factor	Time point	Mean urine cotinine level (ng/mL)	Standard deviation	Mean difference (from baseline)	P value
Age					
18-30	Baseline	260	75	120	-
	6 months	140	55		<0.001
	12 months	120	45	140	<0.001
31-45	Baseline	240	70	110	-
	6 months	130	50		<0.001
	12 months	110	40	130	<0.001
46-60	Baseline	270	85	100	-
	6 months	170	60		<0.001
	12 months	130	50	140	<0.001
Gender					
Male	Baseline	250	80	140	-
	6 months	110	60		<0.001
	12 months	100	40	150	<0.001
Female	Baseline	240	75	120	-
	6 months	120	50		<0.001
	12 months	110	45	130	<0.001

**Figure 2:**
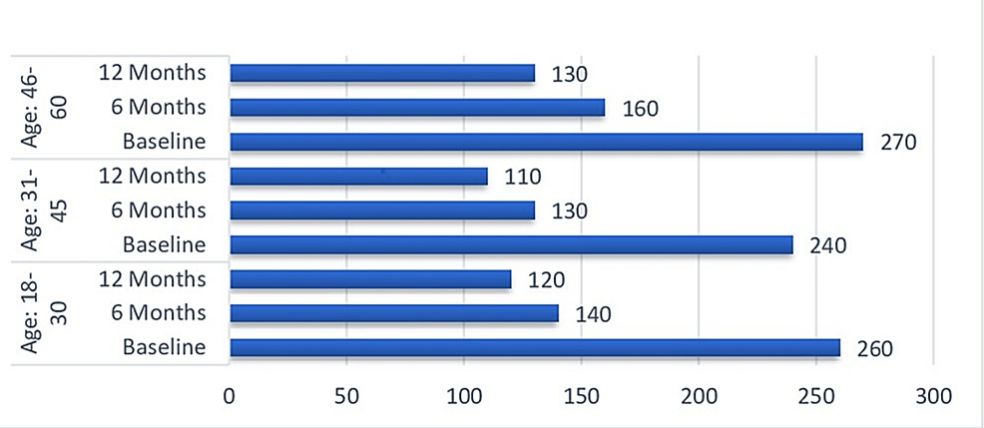
Urine cotinine levels (ng/mL) at baseline and 6 and 12 months postintervention stratified by age group

Initially, the average urine cotinine level among the participants was 250, which was a lot more than the average, and thus, the participants were tobacco consumers on a large scale. The research was conducted after the analysis of the demographic parameters, and it was found that the subjects with the highest number of urine cotinine levels, in the age group of 46-60 with a mean value of 270 ± 85, were the ones with the highest number of urine cotinine levels, as shown in Figure 2.

A huge change was noticeable at all time intervals, with a huge drop in urine cotinine levels from baseline to 6 and 12 months after the intervention. This drop happened by chance because of the CCM. Therefore, there was a large drop in urine cotinine levels from baseline to 6 and 12 months after the intervention. The CCM intervention correlates with socioeconomic factors, which means that a more comprehensive analysis of the impact of the intervention on smoking cessation within the various demographic groups can be done.

Intervention effects on smoking cessation

After the inauguration of CCM among workers, the prevalence of smoking significantly declined among primary health service workers. Initially, the mean urine cotinine level among the participants was 250 ng/mL (SD = 80), which was quite more than the average and suggested that the participants were taking much more nicotine every day. Six months later, the mean urine cotinine level was 150 ± 60, representing a significant (p = ~0.001) difference from the baseline level. This upsurge was reflected at the 12-month follow-up, with the urine cotinine levels getting reduced to 100 ± 40, suggesting that a complete cessation of smoking has been achieved.

Secondary outcome measures

The participants have noticed that the CCM has successfully optimized their lifestyle. Besides, the participants remarked that the levels of urine cotinine, which is the indicator of nicotine in one's body, along with their smoking-related behaviors and attitudes, have undergone a considerable change after the program. The participants' self-reports of having tried at least once to stop smoking have practically doubled as males and females have confronted their smoking habit during the intervention period.

## Discussion

The present study assessed the efficiency of the CCM approach in helping primary healthcare employees quit smoking by investigating the impact of socioeconomic variables on the reduction of urine cotinine levels [5]. Findings showed that the urine cotinine levels were significantly lower at the six-month mark and at the end of the study, between 6 and 12 months after the intervention, proving that most participants had successfully quit smoking.

The significant fall in urine cotinine levels indicated in this study agrees with the findings of previous research on CCM performance. It is a proven tool for helping people deal with multiple chronic conditions, many of which are caused by tobacco use [6,7]. The CCM adopts a patient-oriented approach, and thus, it is possible to design personalized treatment plans besides patient education and support, which are the key factors in successful smoking cessation interventions [5]. Besides, the reported changes in smoking-related behaviours and attitudes of participants also prove the influence of the intervention on smoking cessation in the healthcare setting.

Nevertheless, it is essential to admit that the impact of socioeconomic factors on smoking and quitting habits is significant. It is also a fact that socioeconomic differences, including income, education, and employment status, significantly contribute to the high prevalence of smoking and cessation rates [8,9]. In this study, the segregation of the analysis based on socioeconomic factors revealed varying responses across different demographic groups. Thus, the younger age group (18-30 years) showed a greater decrease in urine cotinine levels than the older age groups (31-45 and 46-60 years) 6 and 12 months after the intervention. This result is in line with the previous studies, which showed that younger people are more responsive to health cessation interventions because of their strong motivation to change their health behaviours and higher perceived susceptibility to health risks [10,11].

Likewise, gender differences were noticed in how the intervention was conducted, with the male participants showing a bigger drop in urine cotinine levels than the female participants. This discovery is remarkable, especially given the fact that studies have shown that the difference in smoking behaviours and quitting results between men and women is significant [12,13]. Gender differences in smoking cessation success are influenced by social norms, nicotine metabolism rates, and the availability of cessation resources [14,15]. Thus, creating smoking cessation interventions that are adapted to the specific needs and preferences of different demographic groups is the key to the success of these interventions and the reduction of health inequalities.

Limitations

One limitation is the absence of a comparison control group. Moreover, the outcomes could just pertain to the specific cohort of primary healthcare professionals who were the focus of the investigation.

## Conclusions

The study provides valuable insights into how CCMs are utilized by healthcare providers to help patients quit smoking. CCM has been proven to be a very efficient health model for smoking cessation. Health behavior models are an effective method for smoking cessation, and their combination with pharmacological methods can also help in enhancing cessation. It is crucial to consider socioeconomic issues when creating and implementing smoking cessation plans, especially for those population groups, to ensure equal access to smoking cessation methods for all population groups. Future research should make use of adapted therapeutics to delineate the mechanisms underlying the consequences of socioeconomic discrepancies on smoking habits and the effects of quitting.
